# Draft Genome Sequence of *Shewanella* sp. ECSMB14102, a Mussel Recruitment-Promoting Bacterium Isolated from the East China Sea

**DOI:** 10.1128/genomeA.00670-15

**Published:** 2015-06-18

**Authors:** Jin-Long Yang, Xing-Pan Guo, Yu-Ru Chen, Wei Gao, De-Wen Ding

**Affiliations:** a Key Laboratory of Exploration and Utilization of Aquatic Genetic Resources, Shanghai Ocean University, Ministry of Education, Shanghai, China; b Marine Ecology Research Center, The First Institute of Oceanography, State Oceanic Administration, Qingdao, China

## Abstract

*Shewanella* sp. ECSMB14102, which promotes recruitment of the mussel *Mytilus coruscus*, was isolated from natural biofilms formed on glass slides submerged in the East China Sea. Here, we present the draft genome sequence, which comprises 4.41 Mb with a G+C content of 52.2%. The genomic information in this strain will contribute to deepening our understanding of bacteria-animal interaction.

## GENOME ANNOUNCEMENT

The interaction between microorganisms and animals has been viewed as an aspect of animal biology that is fundamentally important from development to systems ecology (1). In the marine environment, biofilms exist on almost all exposed substrata and have an important ecological potential to mediate the abiotic and biotic interactions of the host (2). The development of normal animals, such as many marine invertebrates, relies on the partners of microbes (3, 4). The *Shewanella* are aquatic microorganisms with worldwide distribution in marine and freshwater environments (5). More than 50 *Shewanella* genomes have been fully sequenced to date, and many genomes sequenced were selected due to their known energy and bioremediation capabilities, phylogenetic relatedness, and inhabitance of environments (6). *Shewanella* have also been known to modulate the recruitment of some marine invertebrates, including the mussels (7, 8), oysters (9), and sea urchins (10). However, the molecular mechanisms underlying the recruitment of many marine invertebrate species remains unknown. The genetic and genomic approaches provide insight into understanding bacteria-mussel interactions (11).

*Shewanella* sp. ECSMB14102 was isolated from natural biofilms formed on glass slides submerged in the East China Sea (122°77′E; 30°72′N), and the 16S rRNA gene sequences of *Shewanella* sp. ECSMB14102 shared 99% similarity with *Shewanella algae* strain R9 (GenBank accession number KC109735) according to comparison with NCBI databases. The draft genome sequence of the strain ECSMB14102 was sequenced on the Illumina MiSeq platform at the Shanghai Majorbio Pharm Technology Co., Ltd. (Shanghai, China) with a paired-end library. After being trimmed and merged, the reads were *de novo* assembled with GS De Novo Assembler v2.8. Open reading frames (ORFs) were predicted by using the program Glimmer 3.02 (12). All ORFs were then annotated by comparison with NCBI-NR and KEGG using blastp (BLAST 2/2/28+). The tRNA and rRNA genes were predicted by the program tRNAscan-SE v1.3.1 and Barrnap 0.4.2, respectively (13).

The draft genome sequence of the strain ECSMB14102 comprises 4.41 Mb. The largest contig assembled is 221,771 bp. The *N*_50_ and *N*_90_ quality measurements of the contigs were 92,990 bp and 24,960 bp, respectively. The G+C content is 52.2%. The genome contains 3,826 predicted protein-coding sequences (CDSs), 87 tRNA genes for 20 amino acids, and 8 rRNA genes. There was no evidence for a plasmid. The present investigation will provide an entry point to understanding the interaction between the settlement-associated functional genes and the settlement process of the mussel *M. coruscus*.

### Nucleotide sequence accession numbers.

This whole-genome shotgun project has been deposited at DDBJ/EMBL/GenBank under the accession number JWGX00000000. The version described in this paper is the first version, JWGX01000000.
